# Effect of Haemostatic Control Resuscitation on mortality in massively bleeding patients: a before and after study

**DOI:** 10.1111/j.1423-0410.2008.01130.x

**Published:** 2008-11-19

**Authors:** P I Johansson, J Stensballe

**Affiliations:** 1 Department of Clinical Immunology, Blood Bank, and Copenhagen University HospitalCopenhagen, Denmark; 2 Department of Anaesthesia, Centre of Head and Orthopedics, Rigshospitalet, Copenhagen University HospitalCopenhagen, Denmark

**Keywords:** massive bleeding, plasma, platelets, transfusion, thrombelastography

## Abstract

**Background and Objectives:**

Evidence supporting the use of platelets and plasma in resuscitation of massive bleedings is questionable. Current consensus guidelines recommend restrictive use. Our aim was to determine the effect of changing the transfusion practice on 30-day survival in massively bleeding patients.

**Materials and Methods:**

Consecutive adult patients receiving more than 10 units of red blood cells (RBC) within 24 h 2 years prior to (2002–2003) and 2 years after (2005–2006) a change in transfusion practice were included. In 2004, we implemented Haemostatic Control Resuscitation (HCR) with preemptive use of platelets and plasma, administered in transfusion packages, comprising 5 units of RBCs, 5 units of fresh-frozen plasma and 2 units of platelet concentrates (PC), when massive bleeding occurred or upon arrival at the emergency room and thereafter directed by thrombelastography throughout the peri- and postoperative period.

**Results:**

In 2005–2006, the 442 patients received more PCs within 24 h from admission [mean 5·0 (SD 4·2) vs. 1·7 (2·0); *P* < 0·0001] and had a smaller decrease in platelet count during the bleeding episode [91·5 (81·2) vs. 119·7 (100·8) × 10^9^/l; *P* = 0·0025] than the 390 patients treated in 2002–2003. Thirty-day mortality was reduced in 2005–2006 (20·4% vs. 31·5%; *P* = 0·0002) and at 90-day (22·4% vs. 34·6%; *P* < 0·0001) as compared to 2002–2003.

**Conclusions:**

In patients who experience massive bleeding, HCR with platelets and plasma, as guided by thrombelastography, is associated with improved survival. While confirmation from a randomized controlled trial is urgently needed, HCR may be considered in these patients.

## Introduction

Persistent haemorrhage remains a major contributor to mortality in massively transfused patients [1]. A considerable proportion of patients with ongoing bleeding develop coagulopathy. In traumatically injured patients, coagulopathy is already present on admission of the most seriously injured patients and is associated with a poor outcome [2,3]. The complexity of treating massively bleeding patients may result in a suboptimal transfusion therapy further contributing to the poor outcome [4,5]. Traditionally, blood banks have focused on providing compatible blood products of the highest possible quality to meet the requests from clinicians relying on the premise that clinician knowledge of existing transfusion guidelines results in the optimal transfusion therapy for the bleeding patient [6–8]. Existing guidelines, however, advocate early administration of crystalloids and colloids in conjunction with transfusion of red blood cells (RBC). According to the guidelines, fresh-frozen plasma (FFP) and platelets should only be administered when a whole blood volume or more has been substituted and then only in patients with excessive or microvascular bleeding and at best according to conventional laboratory/coagulation analyses. This approach may cause dilution coagulopathy and potentially compromise haemostatic ability further in the most severely bleeding patients [1,9,10]. The recommendations of existing guidelines are based on the result of conventional coagulation assays, such as activated partial thromboplastin time (APTT) and prothrombin time, although these assays poorly correlate with clinically relevant coagulopathies [11,12]. In contrast, cell-based whole blood viscoelastical assays, such as thrombelastography (TEG), provide quantitative information of the haemostatic process and thus give a profile of the haemostatic changes that occur during clotting [13,14]. This may be a better guide for blood component therapy in patients presenting with massive bleeding [9,15–17].

The current transfusion guidelines are now being challenged. Reports based on retrospective observations indicate that plasma and platelets should be administered in a 1 : 1 : 1 ratio (i.e. equal parts of RBC, FFP and platelets) to massively bleeding patients, which simulates the composition of whole blood [4,18]. Recently, we reported on proactive administration of FFP and platelets in patients operated for a ruptured abdominal aortic aneurysm (rAAA), finding that the postoperative transfusion requirements and the 30-day mortality were significantly reduced when patients were treated with platelets and FFP in conjunction with RBCs from the start of resuscitation [19].

In the present study of massively bleeding patients, we evaluated the effect of Haemostatic Control Resuscitation (HCR), entailing preemptive use of platelets and FFP in tailored transfusion packages immediately massive bleeding occurred or upon arrival at the trauma centre with subsequent transfusion therapy directed by the results of TEG analysis throughout the peri- and postoperative period. The hypothesis was that implementing HCR would reduce mortality in severely bleeding patients receiving more than 10 RBCs within 24 h.

## Methods

We undertook a before and after study using historical controls. We included all consecutive adult patients (age ≥ 15 years) who received more than 10 RBCs within 24 h. Patients treated in 2005–2006 were compared to patients treated in 2002–2003 in order to determine patient 30-day and 90-day mortality.

Haemostatic Control Resuscitation for massively bleeding patients was introduced in collaboration between the blood bank and the clinicians based on a joint task force recommendation at our hospital in 2004. The blood bank introduced the following services: (i) transfusion packages comprising 5 units of RBCs stored in saline-adenine-glucose anticoagulated with Macrodex (SAG M) for a maximum of 15 days, 5 units of FFP, and 2 units of platelet concentrates (PC) each pooled from four donors, stored in T-Sol for a maximum of 7 days, to be used from the outset in patients experiencing massive bleeding until the result of the TEG analysis was available in the emergency or operating room; (ii) storage of thawed, ready-to-use FFP in the blood bank for a maximum of 72 h, for use in transfusion packages; (iii) continuous monitoring of the blood transfusion practice in patients receiving more than 10 RBCs within 24 h; (iv) outreach guidance for the treating physicians in order to ensure preemptive use of platelets and FFP in patients with massive bleeding; (v) protocol for monitoring of haemostatic competence with TEG and an intervention algorithm for treatment with plasma and platelets based on the results of the analysis (Appendix); and (vi) educational programme for anaesthesiologists concerning functional haemostasis and TEG.

Thrombelastography was implemented as a routine analysis performed in the blood bank and displayed bedside in the resuscitation rooms of the trauma centre, operation theatres, and in the intensive care units (ICU). The taskforce did not implement any changes in anaesthetic or surgical procedures or in the intensive care management during the study period. Specifically, no changes in the use of endovascular haemostatic treatment were performed and no other type of blood products were used.

The following variables were recorded: gender, age, diagnosis, length of stay (LOS) in the ICU, LOS in hospital, 30- and 90-day follow-up on all cause, overall mortality recorded from the database of the hospital and the Central Office of Civil Registration. Amounts of transfused RBC, FFP, and PC the first 24 h and at 30 days were obtained from the database in the blood bank. Conventional laboratory analyses such as haemoglobin, platelet count, APTT, international normalized ratio (INR) and serum creatinine preceding the first blood transfusion and at 24 h thereafter were obtained from the laboratory database. All data were routinely collected and entered into a study database based on unique personal identity numbers after approval from the Danish Data Protection Agency. The regional ethics committee of Copenhagen approved the waiver of consent, as all procedures were part of standard care.

## Statistics

Data are presented as mean and standard deviation (SD) and analysed by the two-sample *t*-test or χ^2^-test where appropriate. A multivariate logistic regression analysis was used to identify independent risk factors for death as outcome. The Hosmer–Lemeshow goodness-of-fit test was applied to reject the model if *P* < 0·05. Test for collinearity was performed, and collinearity was suspected with a variance proportion > 0·5 and condition index > 10 [20]. We assessed the time to death by Kaplan–Meier analysis using the log-rank test. All statistical analyses were performed using SAS 9·1 (SAS Institute Inc., Cary, NC, USA), and a *P* < 0·05 was considered statistically significant.

## Results

During 2005–2006, 7575 patients received blood transfusions out of 62 434 adult patients admitted to our hospital compared to 7370 adult patients receiving blood transfusion among 58 769 admitted patients in 2002–2003.

In 2005–2006, 442 patients (5·8% of all transfused) received more than 10 RBCs within 24 h compared to 390 patients (5·3% of all transfused), fulfilling the criteria in 2002–2003. There was no significant difference between groups with regard to demographic variables or initial laboratory analyses prior to first blood transfusion with regard to haemoglobin, platelet count, APTT, INR, albumin, bilirubin or creatinine (Table 1). The most frequent cause of surgery was abdominal/vascular followed by cardiothoracic and trauma surgery. Patients treated in 2005–2006 received more PCs within 24 h and at 30 days (Table 2). The FFP : RBC ratio in 2005–2006 was 1 : 1·3 compared to 1 : 1·6 in 2002–2003, *P* < 0·0001. Patients treated in 2005–2006 presented with a smaller decrease in platelet count pre to post as compared to patients treated in 2002–2003 (Table 3). More patients were admitted to the ICU in 2005–2006 [327 (74·0%) vs. 251 (64·4%), *P* = 0·0026] and patients had a longer ICU stay [8·3 days (11·9 days) vs. 6·5 (10·5), *P* = 0·03] and a longer hospital stay (23·5 vs. 19·1, *P* = 0·017) as compared to patients treated in 2002–2003.

**Table 3:** Conventional laboratory measurements prior to start of first transfusion and 24 h after, in 832 patients receiving more than 10 units of RBC within 24 h

	**2005–2006 (*n* = 442)**	**2002–2003 (*n* = 390)**	*P*-value
Pre-haemoglobin (mmol/l)	7·2 (1·5)	6·9 (1·6)	
Post-haemoglobin (mmol/l)	6·7 (0·95)	6·5 (1·0)	
Difference pre to post in haemoglobin (mmol/l)	0·55 (1·7)	0·47 (1·9)	0·67
Pre-creatinine (mmol/l)	0·27 (0·8)	0·25 (0·98)	
Post-creatinine (mmol/l)	0·69 (1·2)	0·88 (1·6)	
Difference pre to post in creatinine (mmol/l)	–0·49 (1·4)	–0·56 (1·6)	0·62
Pre-international normalized ratio	1·3 (0·5)	1·4 (0·7)	
Post-international normalized ratio	1·5 (0·3)	1·5 (0·4)	
Difference in international normalized ratio	–0·10 (0·5)	–0·07 (0·64)	0·70
Pre-APTT (seconds)	33·5 (10·7)	34·4 (8·0)	
Post-APTT (seconds)	37·2 (13·1)	37·9 (13·7)	
Difference pre to post in APTT (seconds)	–3·0 (13·7)	–3·0 (13·0)	0·97
Pre-platelet count (10^9^/l)	208·2 (102·8)	210·6 (122·0)	
Post-platelet count (10^9^/l)	113·5 (55·8)	92·7 (54·5)	
Difference pre to post in platelet count (10^9^/l)	91·5 (81·2)	119·7 (100·8)	0·0025

Data are mean (standard deviation)

Two-sample *t*-test.

**Table 2:** Blood component use during and after initial bleeding episode in 832 patients receiving more than 10 units of red blood cells within 24 h

	**2005–2006 (*n* = 442)**	**2002–2003 (*n* = 390)**	*P*-value
Number of RBC 24 h	18·0 (12·6)	19·2 (15·8)	0·2409
Number of FFP 24 h	13·5 (12·3)	12·1 (15·2)	0·1489
Number of PC 24 h	5·0 (4·2)	1·7 (2·0)	< 0·0001
Number of blood components 24 h	36·5 (27·9)	33·0 (30·4)	0·0816
Number of RBC 30 days	26·6 (24·2)	28·7 (25·5)	0·2263
Number of FFP 30 days	17·5 (17·8)	17·5 (23·5)	0·9489
Number of PC 30 days	7·0 (9·2)	3·4 (7·5)	< 0·0001
Number of blood components 30 days	51·1 (48·0)	49·5 (51·0)	0·6444

RBC, red blood cells; FFP, fresh-frozen plasma; PC, platelet concentrate (four donors).

Data are mean (standard deviation)

Two-sample *t*-test.

**Table 1:** Demographic variables and laboratory results in patients receiving more than 10 units of red blood cells within 24 h in 2005–2006 and 2002–2003

	**2005–2006 (*n* = 442)**	**2002–2003 (*n* = 390)**	**All**	***P*****-value**^a^
Age in years	58·3 (18·5)	58·4 (18·4)	58·3 (18·5)	0·97
Male gender	315 (71·3%)	268 (68·7%)	583 (70·1%)	0·42
Type of admission				
Abdominal-vascular surgery	231(52·3%)	176 (45·1%)	407 (48·9%)	0·1357
Cardiothoracic surgery	83 (18·8%)	87 (22·3%)	170 (20·4%)	
Trauma surgery	60 (13·6%)	61 (15·6%)	121 (14·5%)	
Orthopaedic surgery	38 (8·6%)	27 (6·9%)	65 (7·8%)	
Miscellaneous	22 (5·0%)	24 (6·2%)	46 (5·5%)	
Burn surgery	8 (1·8%)	15 (3·9%)	23 (3·4%)	
Pre-bleeding laboratory analyses				
Plasma albumin (g/l)	30·7 (8·5)	31·1 (6·8)	31·0 (6·2)	0·77
Plasma bilirubin (µmol/l)	14·6 (15·8)	15·3 (12·4)	14·9 (11·1)	0·91
Creatinine (mmol/l)	0·27 (0·8)	0·25 (0·98)	0·26 (0·87)	0·81
APTT (second)	34·4 (8·0)	33·5 (10·7)	34·0 (9·3)	0·48
International normalized ratio	1·3 (0·5)	1·4 (0·7)	1·4 (0·6)	0·11
Platelet count (10^9^/l)	210·6 (122·0)	208·2 (102·8)	209·1 (113·0)	0·83
Pre-bleeding sample time, from blood sampling to first transfusion (h)	13·6 (13·1)	11·8 (12·6)	12·7 (12·9)	0·1437
Post-bleeding sample time, from first transfusion to blood sample (h)	32·6 (12·2)	32·1 (18·8)	33·6 (11·8)	0·4653

Data are mean (standard deviation), if not marked as *n*%.

Two-sample *t*-test and χ^2^-test.

a Difference between 2005–2006 and 2002–2003.

**Fig. 1:**
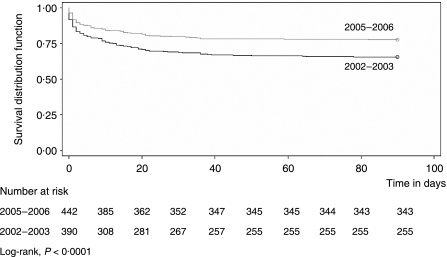
Kaplan–Meier curve for 90-day survival.

All cause mortality at 30 days was 20·4% (*n* = 90) in 2005–2006 compared to 31·5% (*n* = 123) in 2002–2003 (*P* = 0·0002) and 22·4% (*n* = 99) vs. 34·6% (*n* = 135) at 90 days (*P* < 0·0001) (Fig. 1). Each RBC transfused during the first 24 h was independently associated with a 1·05 increased odds ratio for death, whereas an increase in platelet count was independently associated with a 0·994 odds ratio for decrease in mortality per 1 × 10^9^/l count increase (Table 4).

**Table 4:** Multivariate logistic regression analysis of risk factors for 30-day mortality in 832 patients receiving more than 10 RBCs within 24 h

	**Death within 30-days (*n* = 213)**	
	
**Variable**	**Odds ratio (95% CI)**	*P*
Platelet count 24 h after first transfusion (increase 1 × 10^9^/l)	0·99 (0·989 to 0·998)	0·008
Period 2002–2003 vs. 2005–2006	1·6 (1·05 to 2·4)	0·0288
RBC transfusion in 24 h (increase 1 unit)	1·05 (1·03 to 1·08)	< 0·0001
Age (increase 1 year)	1·05 (1·0 to 1·1)	< 0·0001
Cause of surgery		0·0151
Trauma vs. abdominal-vascular surgery	1·4 (0·72 to 2·9)	
Orthopaedic vs. abdominal-vascular surgery	0·15 (0·04 to 0·67)	
Miscellaneous vs. abdominal-vascular surgery	1·9 (0·78 to 4·6)	
Cardiothoracic vs. abdominal-vascular surgery	1·2 (0·72 to 2·0)	
Burn vs. abdominal-vascular surgery	5·0 (1·3 to 20·0)	
Not admitted to the intensive care unit	0·36 (0·16 to 0·82)	0·0151
Time from first transfusion to blood sample (increase 1 h)	0·97 (0·93 to 1·01)	0·0787
Time from blood sample to first transfusion (increase 1 h)	1·02 (0·99 to 1·05)	0·16
Male gender	1·1 (0·7 to 1·7)	0·69
Platelet count prior to first transfusion (increase 1 × 10^9^/l)	0·99 (0·99 to 1·01)	0·59

CI, confidence interval.

Transfusion of fresh frozen plasma and platelets concentrates within 24 h was removed from the model because of suspected collinearity with red blood cell transfusion within 24 h (variance proportion > 0·5 and condition index > 10). (Further data are available on request).

All odds ratios were mutually adjusted for the other variables. Hosmer and Lemeshow goodness-of-fit test, *P* = 0·7381.

A total of 213 patients did not survive 30 days (90 in 2005–2006 vs. 123 in 2002–2003). Among non-survivors, there were no significant differences between groups with regard to age, gender, platelet count prior to the first transfusion, type of surgery, admittance to the ICU, LOS in ICU or hospital LOS, data not shown (available on request). Non-survivors treated in 2005–2006 received more platelet concentrates at 24 h [6·4 (5·8) vs. 1·8 (2·1); *P* < 0·0001] and at 30 days [8·9 (11·6) vs. 3·7 (8·2); *P* = 0·0002] and presented with a higher platelet count 24 h after the bleeding episode [102·3 (52·6) vs. 80·9 (52·1) × 10^9^/l] and a smaller decrease in platelet counts pre to post [62·4 (77·5) vs. 107·5 (91·4) × 10^9^/l; *P* = 0·0248] as compared to patients treated in 2002–2003. Non-survivors treated in 2005–2006 also received more FFP the first 24 h [18·5 (19·3) vs. 13·3 (14·5), *P* = 0·03] than patients treated in 2002–2003.

## Discussion

The implementation of HCR with preemptive platelets and plasma in transfusion packages immediately when massive bleeding occurred or upon arrival at the hospital and thereafter based on TEG results throughout the peri- and postoperative period appears to be associated with reduced mortality in massively bleeding patients. Patients treated in 2005–2006 received more platelet transfusions, which resulted in an improved haemostatic competence as measured by the smaller decrease in platelet count during the bleeding episode. Both the improved platelet count and the treatment period 2005–2006 were independently associated with reduced mortality, after adjustment for relevant variables, such as age, gender, RBC transfusion, admittance to ICU, and cause of surgery.

The results indicate that for massively bleeding patients, adherence to current transfusion guidelines, which are based on observations from controlled blood loss in the elective surgical setting and professional consensus [9,10], may not be optimal. There are several inherent problems with current guidelines, the concentration of platelets is diluted to less than 50% and coagulation factors to about 30% before administering plasma and platelets to massively bleeding patients and then only when microvascular bleeding occur. Moreover, the recommended resuscitation regime applies to all patients, regardless of individual differences in coagulation factor activity, platelet concentration, the dynamics of bleeding, and the extent of tissue damage related to the surgery or trauma, all of which significantly impact patient outcome [21].

Our blood bank therefore introduced thawed, ready–to-use FFP, which enables immediate resuscitation of massive bleeding with platelets and plasma organized in transfusion packages, securing haemostatic competence, as evaluated by TEG, provided the different components are administered in parallel and of equal rate [22].

Another major concern regarding resuscitation of massively bleeding patients is that conventional coagulation analyses are used to identify those patients in need for plasma substitution despite that they only describe isolated fragments of the haemostatic process [11–13]. Furthermore, platelet count alone does not reflect the platelets haemostatic functionality [23]. Instead, TEG, a whole blood analysis measuring the viscoelastical properties of the clot and reflecting the kinetics of thrombin generation, seems more appropriate. TEG has proven superior to conventional coagulation assays and platelet count in that it reduces transfusion requirements in patients undergoing liver and complex cardiac surgery, and TEGs do better predict the need for blood transfusion in trauma patients [15,17,24].

In the present study, the patients treated in 2005–2006 were monitored by TEG after the initial administration of transfusion packages and treated with plasma and platelets according to the TEG results throughout the peri- and postoperative period. This approach may have contributed to the reduced mortality related to clinically important coagulopathies being identified and the treatment monitored. This is further corroborated by Plotkin *et* *al*. who in patients with penetrating injury demonstrated that TEG hypocoagulability, as evidenced by a decreased Angle and clot strength, as opposed to the result of conventional coagulation assays, correlated with increased blood product use [25] and its use is now suggested by recent international guidelines [9,10].

Previous reports on the outcome of patients requiring massive transfusion have documented that development of coagulopathy is associated with poor outcome [2,3,18], and Borgman *et* *al*. [26] demonstrated that early and aggressive replacement of coagulation factors is associated with improved survival by decreasing death from haemorrhage for trauma patients requiring massive transfusion. The mortality was 65% for an FFP : RBC ratio of 1 : 8, 34% for an FFP : RBC ratio of 1 : 2·5, and 19% for an FFP : RBC ratio of 1 : 1·4, which is comparable to the results of the present study where an FFP : RBC ratio of 1 : 1·3 was obtained as well as a mortality of 20% in 2005–2006.

Furthermore, Gonzalez *et* *al*. [27] reported that coagulopathy upon arrival to the hospital was not corrected when the patients entered the ICU despite adherence to pre-ICU massive transfusion protocols. The likely explanation is inadequate pre-ICU haemostatic monitoring and intervention, and more aggressive pre-ICU intervention to correct coagulopathy may be effective in decreasing RBC requirement during ICU resuscitation leading to an improved outcome because of the association between the extent of RBC transfusion and increased mortality.

Important differences exist, however, between these studies and the present. Firstly, the present study describes the effect of implementing a new transfusion practice encompassing transfusion packages and TEG, whereas only retrospective observations without any forms of interventions included have been reported previously. Secondly, the present study encompassed 3–4 times more patients than the studies reported by Borgmann *et* *al*. and Gonzales *et* *al*. further strengthening the results. Also, previous results in massively bleeding patients mainly comes from the trauma setting, whereas the present study included all, unselected patients receiving massive transfusion and it could be speculated whether haemostatic control resuscitation may further reduce mortality also in the trauma population [28,29].

Importantly, not only plasma but also platelets were administered from start of resuscitation in the present study to secure a normal clot strength, which resulted in a higher platelet count 24 h after start of resuscitation. The multivariate analysis demonstrated that an increase in platelet count was independently associated with a reduction in mortality. This is in alignment with retrospective observations [4,18] and with the results from patients operated for rAAA, where platelet count upon arrival in the ICU was independently associated with outcome [19]. Platelets are pivotal for haemostasis, as the activated coagulation factors assemble on the activated platelets to generate the thrombin burst, ultimately leading to the conversion of fibrinogen to fibrin. Furthermore, the platelets interact with fibrin strands to form the clot [30].

The strength of the present study lies in the inclusion of all consecutive patients who experienced massive bleeding. It was conducted at a single centre ensuring the same standard of care in the two comparable groups of equal size and inclusion period. We studied two large groups of massively bleeding patients presented by all surgical specialties, with no change in the observed distribution of surgical specialties from one period to another.

The main limitation of our study relates to the lack of randomization, which was prohibited by the ethics committee due to the inability to achieve informed consent from these patients. Another potential confounder is the possibility that the patients treated in 2005–2006 were less critically ill than those treated in 2002–2003 and that the difference in mortality between groups was merely a result of this and not a consequence of a change in transfusion therapy. However, more patients were admitted to the ICU, and the surgical cause of bleeding, pre-bleeding laboratory analyses and the demographics of the investigated patients underlined that there were no significant differences between the two groups of patients. The only difference observed between the two groups of patients who did not survive was the increased transfusion of platelets and the improved haemostatic competency, as measured by a smaller decrease in platelet counts during the bleeding episode, in patients treated in 2005–2006. Although before and after studies are retrospective in design, all data in our database are prospectively collected. The use of routine audit data, rather than protocolized data collected for research purposes, may affect the results. However, the sample time was not different in the two groups and adjustment for the effect of sample time did not affect our multivariate model.

In conclusion, the present study investigated the effect of a change in transfusion practice, Haemostatic Control Resuscitation, towards use of transfusion packages, early administration of plasma and platelets, and TEG. The combined effect of interventions seems to have had a beneficial effect on survival in this small subpopulation of massively transfused patients; however, it cannot be ruled out that other interventions not accounted for in our analyses may have varied between the two groups. A randomized controlled trial is urgently needed, but until definitive proof is available, the transfusion policy could rely on observational studies as the one presented here.

## Appendix

**Appendix 1:** Thrombelastography (TEG) treatment algorithm for patients with ongoing bleeding

**TEG Parameter**	**Treatment**
R 11–14 min	2 × FFP or 10 ml/kg
R > 14 min	4 × FFP or 20 ml/kg
MA 46–50 mm	1 platelet concentrate
MA < 46 mm	2 platelet concentrates
Angle < 52	2 × FFP or fibrinogen
Ly30 > 8%	Antifibrinolytics

R, R-time, minutes; MA, maximum amplitude; Ly30, lysis in percent 30 min after MA is reached; FFP, fresh-frozen plasma.

One platelet concentrate pooled from the buffy-coat from four donors.
